# Multi-scale fusion convolution network with progressive dilation for real-time salient object detection of surface defects on strip steel

**DOI:** 10.1038/s41598-026-43386-y

**Published:** 2026-05-19

**Authors:** Zhenhua Zhang, Yong Zou, Xiongfeng Liu, Xu Zhang

**Affiliations:** 1 Mechanical Engineering College, Beijing Institute of Petrochemical Technology, Qingyuan North Road, No. 19, Daxing District, Beijing, 102617 Beijing China; 2 School of Computer Science and Engineering, Key Laboratory of New Generation Artificial Intelligence Technology and Its Interdisciplinary Applications, Ministry of Education, Southeast University, No. 2 Southeast University Road, Jiangning Development Zone, Nanjing, 211189 Jiangsu China

**Keywords:** Salient object detection, Multi-scale feature fusion architecture, Progressive dilation, Different receptive fields, Engineering, Materials science, Mathematics and computing

## Abstract

Accurate and efficient salient object detection (SOD) of strip-steel surface defects plays a critical role in maintaining product quality in modern industrial manufacturing. However, existing SOD methods often struggle to balance detection accuracy with inference efficiency, especially when handling complex defect patterns in real-time production environments. To address this challenge, we propose a novel framework named Multi-Scale Fusion Convolution Network with Progressive Dilation (MSFNet-PD), which is specifically designed for real-time salient defect detection. The proposed MSFNet-PD introduces a multi-scale feature fusion architecture that aggregates contextual information from different receptive fields, enabling the model to capture both fine-grained local textures and broader semantic structures of surface defects. In addition, we incorporate a progressive dilation strategy, where dilation rates are gradually increased across convolutional layers. This design enhances the model’s ability to perceive defects of varying sizes without significantly increasing computational cost or degrading feature resolution. Furthermore, MSFNet-PD employs a lightweight backbone and an efficient fusion mechanism, which collectively contribute to faster inference speed, making the network well-suited for deployment in real-world, high-speed strip steel inspection lines. Extensive experiments conducted on the SD-Saliency-900 dataset demonstrate that our method achieves competitive performance in both detection accuracy and processing speed compared with several recent baselines. The promising results affirm the effectiveness of our approach in practical industrial defect inspection scenarios.

## Introduction

In modern industrial production, accurate and efficient detection of strip-steel surface defects is critical for maintaining product quality and minimizing economic losses. With the rapid advancement of computer vision and deep learning techniques, salient object detection (SOD) has emerged as an effective framework for automatically identifying visually significant regions, including surface anomalies in metallic materials. Nevertheless, despite substantial progress in recent years, achieving an appropriate trade-off between detection accuracy and inference efficiency remains challenging, particularly in real-time inspection scenarios characterized by high-resolution inputs and complex defect patterns.

Conventional methods for defect detection on strip steel typically rely on handcrafted features and traditional image processing techniques^1^, which suffer from limited generalizability and poor robustness to noise and illumination changes. More recently, convolutional neural network (CNN)-based approaches have significantly improved detection accuracy by leveraging hierarchical feature representations^2–4^. Among them, encoder-decoder architectures^5,6^ have demonstrated remarkable performance in various segmentation tasks, including salient defect detection.

Despite these advances, two key limitations still hinder the practical deployment of existing SOD models in high-speed industrial environments. First, many models employ heavy backbones or complex attention mechanisms^7^, which increase computational burden and reduce inference speed. Second, while multi-scale representation learning has shown great potential in improving defect localization^6^, most methods rely on fixed dilation or naive fusion strategies, limiting their ability to simultaneously capture fine-grained textures and large-scale contextual information.

To address these challenges, we propose a novel framework named **Multi-Scale Fusion Convolution Network with Progressive Dilation (MSFNet-PD)**, which is specifically designed for real-time salient defect detection. The core idea of MSFNet-PD is to exploit multi-scale contextual information through a lightweight, progressively dilated architecture that can adaptively capture features at varying scales with minimal computational overhead. Specifically, our method introduces a progressive dilation strategy, where dilation rates gradually increase across convolutional layers to enhance the receptive field while preserving spatial resolution. This allows the network to perceive defects of different sizes more effectively compared to traditional fixed-dilation approaches.

Moreover, MSFNet-PD integrates a fast and effective multi-scale feature fusion module, enabling it to jointly learn local details and global semantics without the need for complex upsampling or heavy attention mechanisms. Coupled with a streamlined backbone, the proposed network achieves high inference speed, making it well-suited for real-time inspection tasks in industrial production lines.

To validate the effectiveness of our method, we conduct comprehensive experiments on the SD-Saliency-900 dataset. Results demonstrate that MSFNet-PD achieves competitive performance in terms of both accuracy and speed, surpassing several recent competitive models^8^.

In summary, our main contributions are as follows:

- We propose MSFNet-PD, a novel SOD framework that integrates progressive dilation with multi-scale fusion for efficient and accurate defect detection.
- We design a lightweight architecture that achieves consistent improvement inference speed without compromising detection performance, supporting real-time deployment.
- Extensive experiments on the SD-Saliency-900 dataset demonstrate the strong competitiveness of our approach in terms of efficiency–accuracy balance.

The rest of the paper is organized as follows: Section “Related work” reviews related work on surface defect detection and multi-scale learning. Section “Methods” introduces the proposed MSFNet-PD architecture in detail. Section “Experiment” presents experimental settings and results. Finally, Section “Conclusion” concludes the paper and outlines future directions.

## Related work

### Surface defect detection in industrial applications

Salient object detection (SOD) has attracted increasing attention in industrial quality inspection, especially for detecting surface defects on strip steel. Existing methods can be broadly categorized into classical encoder-decoder architectures, multi-scale feature fusion frameworks, and lightweight real-time models. In this section, we review representative approaches in these categories and highlight their key contributions.

**Encoder-decoder-based architectures** form the backbone of many SOD models. BASNet^9^ adopts a prediction-refinement structure, combining residual learning and a hybrid loss function (BCE + SSIM + IoU) to enhance regional saliency accuracy and boundary clarity. ITSD^10^ employs dual-branch supervision for saliency and contour prediction, leveraging interactive modules and activation control functions to handle boundary ambiguities. U$^2$-Net^7^ introduces a nested U-shaped architecture with RSU blocks to maintain high-resolution multi-scale features, offering both full and lightweight versions.

**Edge-enhanced and defect-specific methods** have been explored for industrial scenarios. EDRNet^11^ alternates between convolutional and residual dense blocks in the decoder, optimizing feature integration through deep supervision and saliency refinement modules (RRS_1D). DACNet^12^ utilizes multi-resolution convolutional branches with attention-guided decoding for dense defect perception. EMIN^13^ enhances edge-localized saliency detection via interactive saliency fusion and dual-branch supervision. EDN^14^ incorporates extreme downsampling in the encoder and scale-aware fusion in the decoder, offering a lightweight version (EDN-Lite) for real-time applications.

**Real-time and lightweight segmentation networks** are increasingly adopted in industrial SOD. BiSeNet^15^ employs spatial and context paths along with attention and fusion modules for fast and accurate semantic segmentation. LEDNet^16^ reduces computation with asymmetric structures and efficient decoder designs (APN), achieving high-speed inference. CGNet^17^ emphasizes joint local-context learning in a deep-and-thin architecture for edge device deployment. CSNet^18^ integrates gOctConv and dynamic weight decay to reduce feature redundancy while enhancing multi-scale fusion.

**Emerging attention-based and biologically inspired methods** further enrich the SOD landscape. SAMNet^19^ fuses multi-scale features via stereo attention for mobile-friendly SOD. HVPNet^20^ simulates primate visual perception hierarchies and leverages dropout-based attention for robust lightweight detection. FSMI-Net^21^ compresses channels before multi-scale interaction, using hybrid losses and deep supervision to enhance accuracy in optical remote sensing imagery.

In summary, while many approaches excel in detection accuracy or inference speed, achieving a fine balance between real-time performance and saliency precision in industrial defect scenarios remains a challenging task. Motivated by this, recent works such as **MINet**^22^ seek to fuse multi-scale features in lightweight architectures under deep supervision to push the frontier of real-time salient defect detection.

### Multi-scale feature learning

Capturing multi-scale contextual information is essential for accurate defect localization, as defects can vary dramatically in size, shape, and contrast. One common approach is to use image pyramids or dilated convolutions to expand the receptive field. The atrous spatial pyramid pooling (ASPP) module, as introduced in DeepLab^6^, aggregates features from multiple dilation rates and has been widely adopted in segmentation networks. Similarly, pyramid pooling modules (PPM) in PSPNet^23^ allow models to collect contextual cues at different spatial scales.

More recent efforts have moved toward integrating multi-scale fusion directly into network architectures. For instance, U$^2$-Net^7^ employs a nested U-structure to recursively aggregate deep and shallow features. C$^2$FNet^24^ introduces coarse-to-fine contextual integration, which improves boundary precision while keeping the model efficient. However, these designs may still involve repeated upsampling and attention operations, leading to increased memory usage and slower inference.

In parallel, the vision community has sought to build real-time capable salient object detection (SOD) frameworks for resource-constrained environments. Lightweight backbones and efficient fusion mechanisms have been explored to reduce the model size and latency^25^. Transformer-based backbones such as Pyramid Vision Transformer (PVT)^8^, TransUNet^26^, and Swin Transformer^27^ have demonstrated strong performance by modeling long-range dependencies, yet their high computational complexity limits real-time deployment without further optimization.

The concept of progressive dilation has recently gained attention as a method for adaptively enlarging the receptive field across network layers without significantly degrading resolution. By incrementally increasing dilation rates layer by layer, networks can maintain feature granularity while enhancing semantic capture. However, the full potential of progressive dilation remains underexplored in industrial defect inspection, particularly in combination with lightweight and real-time feature fusion designs.

Our work builds on these advances by introducing a unified architecture-MSFNet-PD-that combines multi-scale fusion and progressive dilation in a compact, efficient manner. Unlike previous methods that rely on heavy modules or fixed-scale dilations, our design provides a dynamic receptive field growth with minimal computational overhead, enabling accurate and real-time detection of diverse surface defects.

## Methods

### Task definition

Given an input image $I \in \mathbb {R}^{3 \times H \times W}$ and its corresponding pixel-wise ground truth annotation $G \in \{0,1\}^{H \times W}$, the goal of this task is to learn a mapping function:

1 $$\begin{aligned} f_{\theta }: \mathbb {R}^{3 \times H \times W} \rightarrow [0,1]^{H \times W} \end{aligned}$$

where $f_{\theta }(I) = S$, and $S \in [0,1]^{H \times W}$ denotes the predicted saliency map, representing the probability of each pixel belonging to a defect region.

To achieve this, an encoder-decoder network architecture is adopted. The model is trained by minimizing the discrepancy between the predicted saliency map $S$ and the ground truth $G$, using a hybrid loss function defined as:

2 $$\begin{aligned} \mathcal {L}_{\text {total}} = \sum _{i=1}^{5} \left( \mathcal {L}_{\text {BCE}}(S_i, G) + \mathcal {L}_{\text {SSIM}}(S_i, G) \right) \end{aligned}$$

where $S_i$ denotes the prediction at the $i$-th decoding stage, and deep supervision is applied to improve the accuracy and boundary preservation of defect detection.

### Model architecture

**Fig. 1:**
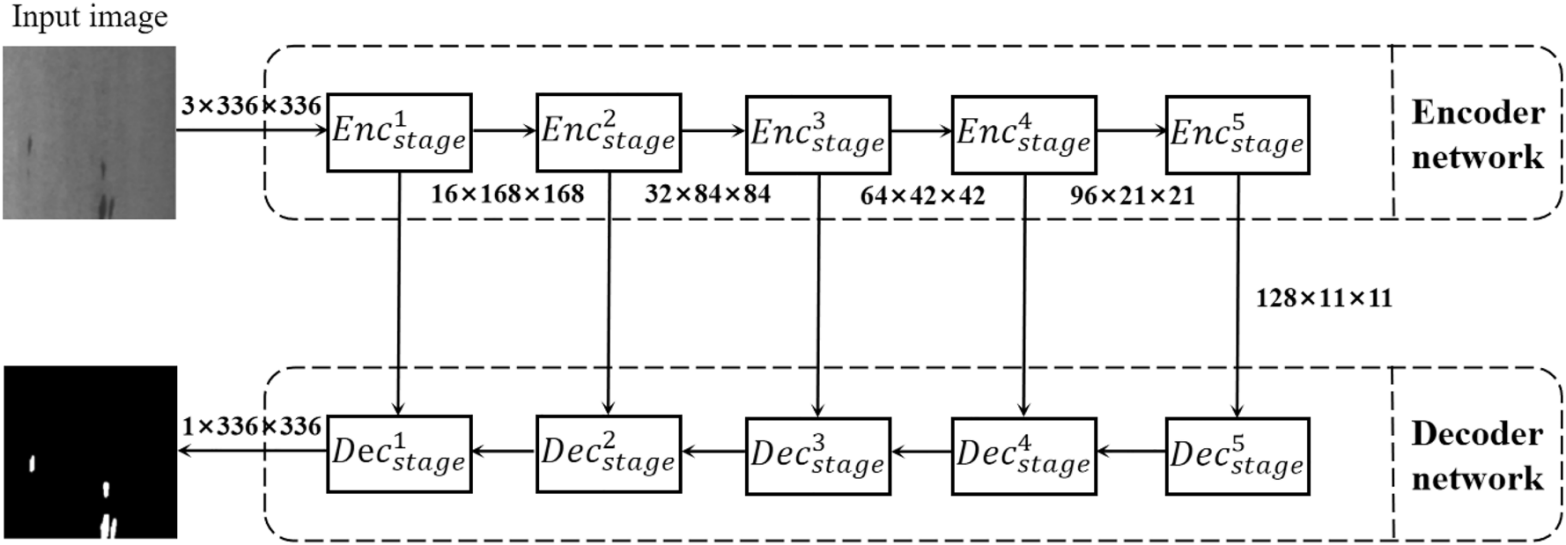
The architecture of MSFNet-PD.

As shown in Fig. 1, this paper provides a detailed introduction to the model architecture based on Multi-Scale Fusion Convolution Network with Progressive Dilation (MSFNet-PD). The architecture employs an encoder-decoder structure to achieve feature extraction and semantic segmentation of input images. The core of the model lies in the multi-scale feature extraction and feature fusion mechanism, which can effectively handle complex image tasks.

### Encoder part

This section details the encoder design and multi-scale feature extraction strategy of MSFNet-PD.

The encoder part gradually reduces the spatial resolution of the feature maps through multiple stages while increasing the number of channels to extract higher-level semantic information.

#### Encoder$_{Stage}^1$

The input image $x$ is processed by a combination module of a convolutional layer, batch normalization (Batch Normalization), and ReLU activation function, as follows:

- **Convolutional Layer**: A convolutional kernel of size $3 \times 3$ with stride $s = 2$ and padding $p = 1$ is used to map the input image’s channels from 3 to 16. The convolution operation can be expressed as:
  $$\text {feat}_1 = \text {Conv}(x; \text {kernel} = 3, \text {stride} = 2, \text {padding} = 1)$$
- **Batch Normalization**: Batch normalization is applied to the feature map after convolution to stabilize the training process and accelerate convergence:
  $$\text {feat}_1 = \text {BatchNorm}(\text {feat}_1)$$
- **ReLU Activation Function**: The ReLU activation function is applied to the feature map after batch normalization to introduce non-linearity:
  $$\text {feat}_1 = \text {ReLU}(\text {feat}_1)$$

After this stage, the size of the feature map is halved, and the number of channels increases to 16.

#### Encoder$_{Stage}^2$

**Fig. 2:**
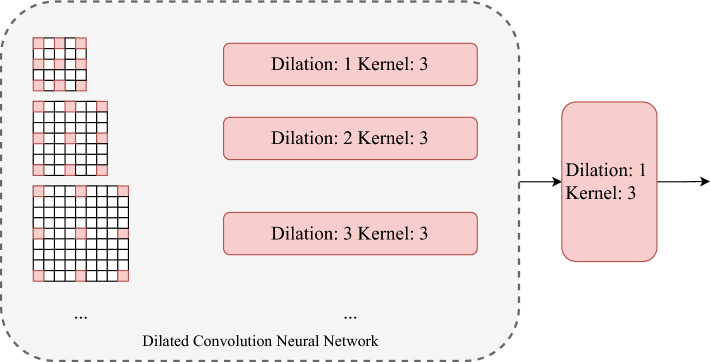
The architecture of MSDC.

- **Depthwise Separable Convolution (DSConv3x3)**: Depthwise separable convolution is performed on the output $\text {feat}_1$ from Stage 1. Depthwise separable convolution decomposes standard convolution into depthwise convolution (per-channel convolution) and pointwise convolution (1x1 convolution):
  - **Depthwise Convolution**: A $3 \times 3$ convolutional kernel with stride $s = 2$ and padding $p = 1$ is used to convolve each channel separately, maintaining the number of channels (16):
    $$\text {feat}_2 = \text {DepthwiseConv}(\text {feat}_1; \text {kernel} = 3, \text {stride} = 2, \text {padding} = 1)$$
  - **Pointwise Convolution**: A $1 \times 1$ convolutional kernel is used to increase the number of channels from 16 to 32:
    $$\text {feat}_2 = \text {PointwiseConv}(\text {feat}_2; \text {kernel} = 1)$$
- **Multi-Scale Dilated Convolution Module(MSDC)**: As shown in Fig. 2, Multi-scale dilated convolution is performed on the output $\text {feat}_2$ from the depthwise separable convolution, as follows:
  - **Multi-Scale Feature Extraction**: Multi-scale features are extracted using convolutional kernels with different dilation rates:
    $$\begin{aligned} \text {feat}\_{\text {e1}}&= \text {Conv}(\text {feat}_2; \text {kernel} = 3, \text {dilation} = 1, \text {padding} = 1) \\ \text {feat}\_{\text {e2}}&= \text {Conv}(\text {feat}_2; \text {kernel} = 3, \text {dilation} = 2, \text {padding} = 2) \\ \text {feat}\_{\text {e3}}&= \text {Conv}(\text {feat}_2; \text {kernel} = 3, \text {dilation} = 3, \text {padding} = 3) \\ \text {feat}\_{\text {e4}}&= \text {Conv}(\text {feat}_2; \text {kernel} = 3, \text {dilation} = 4, \text {padding} = 4) \end{aligned}$$
  - **Feature Fusion**: The multi-scale features are concatenated along the channel dimension to obtain a feature map with $4 \times 32 = 128$ channels:
    $$\text {feat}\_{\text {cat}} = \text {Concat}(\text {feat}\_{\text {e1}}, \text {feat}\_{\text {e2}}, \text {feat}\_{\text {e3}}, \text {feat}\_{\text {e4}})$$
  - **Channel Compression**: A $1 \times 1$ convolutional kernel is used to compress the concatenated feature map back to 32 channels:
    $$\text {feat}_2 = \text {Conv}(\text {feat}\_{\text {cat}}; \text {kernel} = 1)$$

After this stage, the size of the feature map is halved again, and the number of channels increases to 32.

#### Encoder$_{Stage}^3$

- **Depthwise Separable Convolution (DSConv3x3)**: Depthwise separable convolution is performed on the output from Stage 2 to increase the number of channels from 32 to 64 with a stride of 2.
- **Multi-Scale Dilated Convolution Module(MSDC)**: Multi-scale dilated convolution is applied to the output of the depthwise separable convolution to extract and fuse multi-scale features, resulting in a feature map with 64 channels and a size reduction by half.

#### Encoder$_{Stage}^4$

- **Depthwise Separable Convolution (DSConv3x3)**: Depthwise separable convolution is performed on the output from Stage 3 to increase the number of channels from 64 to 96 with a stride of 2.
- **Multi-Scale Dilated Convolution Module(MSDC)**: Multi-scale dilated convolution is applied to the output of the depthwise separable convolution to extract and fuse multi-scale features, resulting in a feature map with 96 channels and a size reduction by half.

#### Encoder$_{Stage}^5$

- **Depthwise Separable Convolution (DSConv3x3)**: Depthwise separable convolution is performed on the output from Stage 4 to increase the number of channels from 96 to 128 with a stride of 2.
- **Multi-Scale Dilated Convolution Module(MSDC)**: Multi-scale convolution is applied to the output of the depthwise separable convolution to extract and fuse multi-scale features, resulting in a feature map with 128 channels and a size reduction by half.

### Decoder part

This section describes the decoder structure and feature fusion mechanism.

The decoder part gradually restores the spatial resolution of the feature maps through multiple stages while reducing the number of channels. Feature fusion is employed to achieve semantic segmentation of the input image.

#### Decoder$_{Stage}^5$

- **Depthwise Separable Convolution (DSConv3x3)**: Depthwise separable convolution is performed on the output from the encoder’s Stage 5, including:
  - Depthwise convolution using a $3 \times 3$ convolutional kernel with dilation rate 2, maintaining 128 channels:
    $$\text {feat}_5 = \text {DepthwiseConv}(\text {feat}_5; \text {kernel} = 3, \text {dilation} = 2)$$
  - Pointwise convolution using a $1 \times 1$ convolutional kernel to reduce the number of channels from 128 to 96:
    $$\text {feat}_{5} = \text {PointwiseConv}(\text {feat}_5; \text {kernel} = 1)$$
- **Upsampling**: The feature map obtained in the previous step is upsampled using bilinear interpolation to match the size of the feature map from the encoder’s Stage 4:
  $$\text {feat}_5^{'} = \text {Upsample}(\text {feat}_5, \text {size} = \text {feat}_4.\text {size})$$

#### Decoder$_{Stage}^4$

- **Feature Fusion**: The upsampled feature map from the decoder’s Stage 5 is added to the output feature map from the encoder’s Stage 4:
  $$\text {feat}_4 = \text {feat}_4 + \text {feat}_{5}^{'}$$
- **Depthwise Separable Convolution (DSConv3x3)**: Depthwise separable convolution is performed on the fused feature map, including:
  - Depthwise convolution using a $3 \times 3$ convolutional kernel with dilation rate 2, maintaining 96 channels:
    $$\text {feat}_4 = \text {DepthwiseConv}(\text {feat}_4; \text {kernel} = 3, \text {dilation} = 2)$$
  - Pointwise convolution using a $1 \times 1$ convolutional kernel to reduce the number of channels from 96 to 64:
    $$\text {feat}_4 = \text {PointwiseConv}(\text {feat}_4; \text {kernel} = 1)$$
- **Upsampling**: The feature map obtained in the previous step is upsampled using bilinear interpolation to match the size of the feature map from the encoder’s Stage 3:
  $$\text {feat}_{4}^{'} = \text {Upsample}(\text {feat}_4, \text {size} = \text {feat}_3.\text {size})$$

#### Decoder$_{Stage}^3$

- **Feature Fusion**: The upsampled feature map from the decoder’s Stage 4 is added to the output feature map from the encoder’s Stage 3:
  $$\text {feat}_3 = \text {feat}_3 + \text {feat}_4^{'}$$
- **Depthwise Separable Convolution (DSConv3x3)**: Depthwise separable convolution is performed on the fused feature map, including:
  - Depthwise convolution using a $3 \times 3$ convolutional kernel with dilation rate 2, maintaining 64 channels:
    $$\text {feat}_3 = \text {DepthwiseConv}(\text {feat}_3; \text {kernel} = 3, \text {dilation} = 2)$$
  - Pointwise convolution using a $1 \times 1$ convolutional kernel to reduce the number of channels from 64 to 32:
    $$\text {feat}_3 = \text {PointwiseConv}(\text {feat}_3; \text {kernel} = 1)$$
- **Upsampling**: The feature map obtained in the previous step is upsampled using bilinear interpolation to match the size of the feature map from the encoder’s Stage 2:
  $$\text {feat}_3^{'} = \text {Upsample}(\text {feat}_3, \text {size} = \text {feat}_2.\text {size})$$

#### Decoder$_{Stage}^2$

- **Feature Fusion**: The upsampled feature map from the decoder’s Stage 3 is added to the output feature map from the encoder’s Stage 2:
  $$\text {feat}_2 = \text {feat}_2 + \text {feat}_3^{'}$$
- **Depthwise Separable Convolution (DSConv3x3)**: Depthwise separable convolution is performed on the fused feature map, including:
  - Depthwise convolution using a $3 \times 3$ convolutional kernel with dilation rate 2, maintaining 32 channels:
    $$\text {feat}_2 = \text {DepthwiseConv}(\text {feat}_2; \text {kernel} = 3, \text {dilation} = 2)$$
  - Pointwise convolution using a $1 \times 1$ convolutional kernel to reduce the number of channels from 32 to 16:
    $$\text {feat}_2 = \text {PointwiseConv}(\text {feat}_2; \text {kernel} = 1)$$
- **Upsampling**: The feature map obtained in the previous step is upsampled using bilinear interpolation to match the size of the feature map from the encoder’s Stage 1:
  $$\text {feat}_2^{'} = \text {Upsample}(\text {feat}_2, \text {size} = \text {feat}_1.\text {size})$$

#### Decoder$_{Stage}^1$

- **Feature Fusion**: The upsampled feature map from the decoder’s Stage 2 is added to the output feature map from the encoder’s Stage 1:
  $$\text {feat}_1 = \text {feat}_1 + \text {feat}_2^{'}$$
- **Depthwise Separable Convolution (DSConv3x3)**: Depthwise separable convolution is performed on the fused feature map, including:
  - Depthwise convolution using a $3 \times 3$ convolutional kernel with dilation rate 2, maintaining 16 channels:
    $$\text {feat}_1 = \text {DepthwiseConv}(\text {feat}_1; \text {kernel} = 3, \text {dilation} = 2)$$
  - Pointwise convolution using a $1 \times 1$ convolutional kernel to maintain 16 channels:
    $$\text {feat}_1^{'} = \text {PointwiseConv}(\text {feat}_1; \text {kernel} = 1)$$

### Output part

Finally, the overall output formulation and deep supervision strategy are introduced.

At each stage of the decoder (Stage 1, Stage 2, Stage 3, Stage 4, and Stage 5), the ‘ConvOut‘ module is used to generate the output. The operations of the ‘ConvOut‘ module are as follows:

- **Dropout**: Dropout is applied to the input feature map with a dropout rate of 0.1 to prevent overfitting:
  $$\text {feat} = \text {Dropout}(\text {feat}; p = 0.1)$$
- **Convolution**: A $1 \times 1$ convolutional kernel is used to reduce the number of channels to 1:
  $$\text {output} = \text {Conv}(\text {feat}; \text {kernel} = 1)$$
- **Sigmoid Activation Function**: The Sigmoid activation function is applied to the feature map after convolution to restrict the output values within the range [0,1] for binary classification tasks:
  $$\text {output} = \sigma (\text {output})$$
- **Upsampling**: The output from each stage is upsampled using bilinear interpolation to match the size of the input image, resulting in the final multi-scale output:
  $$\text {output} = \text {Upsample}(\text {output}, \text {size} = \text {input}.\text {size})$$

The model effectively extracts multi-scale features and achieves high-quality semantic segmentation of the input image through the encoder-decoder structure combined with depthwise separable convolution and multi-scale dilated convolution mechanisms. This architecture performs well in handling complex image tasks, demonstrating high efficiency and accuracy.

The five outputs of the decoder part, $\text {feat}_1^{'}$, $\text {feat}_2^{'}$, $\text {feat}_3^{'}$, $\text {feat}_4^{'}$, and $\text {feat}5^{'}$, are processed by the output part to generate the set $\{S_i\}_{i=1}^5 = \{output_1,output_2,output_3,output_4,output_5\}$.

### Loss function

To enhance the training stability and improve the boundary accuracy of defect localization, we adopt a hybrid loss function combining the Binary Cross-Entropy (BCE) loss and the Structural Similarity (SSIM) loss^28^. This design follows the deep supervision strategy, where predictions from all decoder stages are supervised simultaneously.

Let $\{S_i\}_{i=1}^5$ denote the predicted saliency maps at different decoder stages and $G \in \{0,1\}^{H \times W}$ be the ground truth binary mask. The total loss function is defined as:

3 $$\begin{aligned} \mathcal {L}_{\text {total}} = \sum _{i=1}^{5} \left( \mathcal {L}_{\text {BCE}}(S_i, G) + \mathcal {L}_{\text {SSIM}}(S_i, G) \right) , \end{aligned}$$

where $\mathcal {L}_{\text {BCE}}$ penalizes pixel-wise differences between prediction and ground truth, and $\mathcal {L}_{\text {SSIM}}$ emphasizes the structural similarity, which helps preserve boundary information.

The BCE loss is defined as:

4 $$\begin{aligned} \mathcal {L}_{\text {BCE}}(S, G) = - \frac{1}{HW} \sum _{x=1}^{H} \sum _{y=1}^{W} \left[ G_{x,y} \log (S_{x,y}) + (1 - G_{x,y}) \log (1 - S_{x,y}) \right] , \end{aligned}$$

and the SSIM loss is computed as:

5 $$\begin{aligned} \mathcal {L}_{\text {SSIM}}(S, G) = 1 - \text {SSIM}(S, G), \end{aligned}$$

where SSIM measures the perceptual similarity between the predicted map and the ground truth.

This hybrid loss encourages both pixel-level correctness and structural consistency, making it especially suitable for detecting small and irregular surface defects with fine boundaries.

## Experiment

### Dataset description

To evaluate the effectiveness of the proposed **model**, experiments are conducted on the challenging SD-Saliency-900 dataset^11^, which is specifically designed for strip steel surface defect detection. The SD-Saliency-900 dataset consists of 1,800 pixel-wise annotated RGB images, categorized into three defect types: inclusion, patches, and scratches. Each defect type includes 300 training images and 300 testing images, yielding a total of 900 images per set. The original image resolution is $200 \times 200$. To improve model robustness and prevent overfitting, horizontal flipping is applied to augment the training data, resulting in 1,620 training samples in total. All training and testing images are resized to $368 \times 368$ during preprocessing, and random cropping of $336 \times 336$ is used during training to enhance generalization.

### Implementation details

The proposed MSFNet-PD was implemented using the PyTorch framework. The training and inference benchmarking of MSFNet-PD were conducted on a high-performance workstation equipped with an Intel CPU and a single NVIDIA GeForce RTX 3090 GPU (24GB video memory). For controlled comparison, MINet was re-implemented based on its official open-source code and evaluated under the same RTX 3090 environment. During the training phase, we applied data augmentation strategies to enhance the model’s generalization capability. Specifically, the input images were first rescaled to 368 $\times$ 368 and subsequently randomly cropped to a resolution of 336 $\times$ 336. The model was optimized using the Adam optimizer with parameters set to $\beta _1=0.9$, $\beta _2=0.999$, and $\epsilon =10^{-8}$. The batch size was set to 32, and the initial learning rate was initialized at $4 \times 10^{-3}$ with no weight decay. The network was trained for approximately 1,800 epochs until convergence. For the loss function, we adopted a deep supervision strategy across five scales ($d_1$ to $d_5$). The total loss is defined as the sum of the hybrid losses from all five stages, where each stage combines the Binary Cross-Entropy (BCE) loss and the Structural Similarity (SSIM) loss with equal weights (1:1), as formulated in Equation (3). No additional learning rate scheduler was employed during the training process.

Such a hybrid formulation has been widely adopted in recent SOD methods to preserve fine structural details. While the Binary Cross-Entropy (BCE) loss provides pixel-level supervision to ensure classification correctness, the SSIM loss focuses on structural consistency by evaluating local luminance, contrast, and structural similarity between the predicted saliency map and the ground truth. This combination is particularly beneficial for strip-steel surface defect detection, where fine boundaries and subtle structural variations are critical for accurate localization.

### Evaluation metrics

To comprehensively assess the detection accuracy and computational efficiency of different methods, the following evaluation metrics are employed:

- **Mean Absolute Error (MAE)**: Measures the average absolute difference between the predicted saliency map and the ground truth.
- **Weighted F-measure (WF)**: A variant of the traditional F-measure that incorporates spatial weights to emphasize foreground precision and recall.
- **Structure Measure (SM)**: Captures the structural similarity between the predicted saliency map and the ground truth.

To assess computational efficiency, the following metrics are also reported:

- **Parameters (Param)**: Total number of learnable parameters in the model (in millions).
- **FLOPs**: Floating-point operations required during inference (in giga operations).
- **Speed (FPS)**: Inference speed measured in frames per second (FPS) on both GPU and CPU platforms. For fair benchmarking, the FPS of MSFNet-PD and MINet (Re-implement) was measured on an NVIDIA RTX 3090 GPU with batch size = 1 and an input resolution of 336$\times$336.

### Baselines

To ensure a comprehensive evaluation, we compare MSFNet-PD with representative SOD and lightweight segmentation models, which are briefly introduced as follows.

**MINet** proposes a lightweight multi-scale interaction network that utilizes MI modules with DWConv and PWConv to extract and fuse multi-scale features, forming a real-time backbone network based on an encoder-decoder architecture and deep supervision strategy for low-latency, high-precision salient defect detection on strip steel surfaces^22^.

**BASNet** adopts a prediction-refinement architecture, where the prediction module is a U-Net-like network that generates saliency maps and the refinement module optimizes results via residual learning^9^. A hybrid loss combining BCE, SSIM, and IoU improves regional segmentation quality and boundary clarity.

**CPD** accelerates inference by discarding shallow high-resolution features in a partial decoder and optimizes deep features using generated saliency maps^29^. A dual-branch network produces initial saliency and refined features, integrating global attention and contextual modules for accurate and fast salient object detection.

**PoolNet** is based on a U-shaped architecture and designs GGM and FAM modules. GGM passes global guidance via PPM and GGFs, while FAM addresses the fusion of coarse features with multi-scale features^30^. An edge detection branch enhances boundary details, improving real-time saliency detection.

**F**$^3$**Net** consists of CFM and CFD modules, where CFM selectively aggregates multi-level features to denoise and sharpen boundaries, and CFD iteratively refines features^31^. A proposed PPA loss directs attention to fine details for high-quality saliency map generation.

**ITSD** adopts an encoder-decoder structure with dual branches to learn salient regions and contours^10^. Feature interaction is conducted via the FCF module, and the ACT function is proposed to handle boundary-hard samples, enhancing performance using saliency and contour supervision.

**U**$^2$**-Net** employs a nested U-structure with RSU blocks in each stage to extract multi-scale features while preserving resolution^7^. It is independent of pre-trained classifiers and provides both standard and lightweight versions for real-time high-precision salient object detection.

**SUCA** includes PDC and MACF modules, where PDC uses parallel dilated convolutions to enlarge the receptive field, and MACF cascades channel attention blocks to integrate inter-layer and inter-channel information, improving performance through multi-level loss functions^32^.

**EDN** employs EDB for extreme downsampling to learn global views and SCPC modules in the decoder to recover fine details by fusing multi-scale features^14^. Based on VGG16, a lightweight EDN-Lite version is proposed for improved localization accuracy and real-time performance.

**EDRNet** extracts multi-scale defect features in the encoder via CNNs with attention, and alternates CWB and RDB modules in the decoder to integrate features^11^. The RRS_1D module optimizes saliency maps under deep supervision for strip steel surface defect detection.

**DACNet** adopts a U-shaped structure where the encoder leverages multi-resolution convolutional branches and CFI units to extract and fuse multi-scale features^12^. The decoder integrates features under dense attention guidance and employs deep supervision to enhance defect detection performance.

**EMIN** builds on a U-shaped architecture where the encoder extracts multi-scale features and the decoder aggregates them via interactive and edge-guided saliency fusion^13^. Edge and saliency branches with deep supervision improve the accuracy and robustness of defect detection.

**BiSeNet** utilizes a dual-path structure where the spatial path preserves spatial information and the context path enlarges the receptive field^15^. FFM and ARM are designed to fuse features for fast and accurate semantic segmentation.

**LEDNet** adopts an asymmetric encoder-decoder structure, introducing channel split and shuffle in the encoder and using APN in the decoder to reduce computation complexity while achieving real-time high-accuracy semantic segmentation^16^.

**CGNet** centers on the CG module for learning joint local-context features^17^. Following a “deep and thin” design and input injection mechanism, it achieves lightweight and accurate semantic segmentation.

**CSNet** builds on the gOctConv module and a dynamic weight decay scheme, efficiently leveraging multi-scale features and reducing redundancy to achieve low-parameter, high-accuracy salient object detection^18^.

**SAMNet** incorporates a SAM module using stereo attention to fuse multi-scale features within a lightweight encoder-decoder network, reducing parameters and computation for mobile salient object detection^19^.

**HVPNet** draws inspiration from primate visual systems via an HVP module that simulates hierarchical perception learning, combined with attention mechanisms and dropout strategies to achieve lightweight and precise salient object detection^20^.

**FSMINet** is based on the FSM module, which compresses channels before extracting multi-scale features^21^. Using an encoder-decoder structure with deep supervision and hybrid loss functions, it enables lightweight and high-accuracy salient object detection in optical remote sensing imagery.

### The effectiveness of MSFNet-PD

**Table 1:** Performance of each method.

**Methods**	**Param**	**FLOPs**	**Speed(FPS)**	**MAE** $\downarrow$	**WF** $\uparrow$	**SM** $\uparrow$
BASNet	87.06	127.40	57	0.0152	0.9092	0.8267
CPD	29.23	59.43	137	0.0211	0.8192	0.7749
PoolNet	52.51	117.00	70	0.0215	0.8476	0.7456
F$^3$Net	25.54	16.43	217	0.0150	0.9101	0.8401
ITSD	26.07	15.94	208	0.0153	0.9012	0.8099
U$^2$Net	44.01	58.77	79	0.0143	0.9071	0.8134
SUCA	115.58	56.75	110	0.0125	0.9191	0.8425
EDN	21.83	58.01	60	0.0149	0.9115	0.8308
EDRNet	39.31	42.14	76	0.0130	0.9225	0.8417
DACNet	98.39	142.71	54	0.0118	0.9275	0.8464
EMINet	99.13	139.71	52	0.0119	0.9253	0.8447
BiSeNet	13.24	7.76	709	0.0178	0.8803	0.7848
LEDNet	0.92	2.95	408	0.0182	0.8782	0.7730
CGNet	0.49	1.78	605	0.0172	0.8825	0.7854
CSNet	0.14	0.72	495	0.0309	0.7659	0.7026
SAMNet	1.33	0.54	621	0.0278	0.8161	0.7082
HVPNet	1.23	1.12	567	0.0271	0.8231	0.7110
FSMINet	3.56	11.79	62	0.0178	0.8899	0.8008
MINet	0.28	0.33	721	0.0169	0.8945	0.8115
MINet$_{Re-implement}$	0.28	0.30	1028	0.0179	0.8873	0.9176
MSFNet-PD	**1.59**	**1.33**	2095	**0.0174**	**0.8870**	**0.9202**

As can be seen from the Table 1, there is a significant variation in speed (FPS) among different methods, ranging from 52 FPS to 2095 FPS. The slowest method is EMINet, with only 52 FPS, while the fastest is MSFNet-PD, achieving 2095 FPS. This reflects substantial architectural differences in computational efficiency across methods.

Relationship Between Speed, Parameters, and FLOPs: Generally, larger parameter counts and higher FLOPs indicate more complex network architectures and greater computational demands, leading to relatively slower speeds. For example, BASNet has high parameter counts and FLOPs, resulting in a speed of 57 FPS. In contrast, methods with lower parameter counts and FLOPs, such as LEDNet and CGNet, achieve faster speeds of 408 FPS and 605 FPS, respectively. However, MSFNet-PD, despite not having the lowest parameter count or FLOPs, achieves the highest speed. This suggests that its architectural design enables significant speed improvements while maintaining reasonable performance.

Balancing Speed with Other Performance Metrics: In practical applications, a trade-off must be made between speed and performance metrics (e.g., MAE, WF, SM). For instance, MINet achieves a fast speed of 721 FPS, but its MAE, WF, and SM metrics are slightly inferior compared to slower methods like SUCA and EDRNet. On the other hand, slower methods such as DACNet and EMINet exhibit relatively better performance metrics. This demonstrates that when selecting a network method, it is necessary to consider both speed and performance based on specific application requirements to achieve optimal results.

Characteristics of the Fastest Method, MSFNet-PD: MSFNet-PD stands out with a clear advantage in speed, far surpassing other methods. This indicates that the method employs an efficient network architecture and optimization strategy, enabling rapid data processing and inference. At the same time, it also performs well in metrics such as MAE, WF, and SM, showing that it does not sacrifice performance excessively in pursuit of speed. Such a method, which achieves a good balance between speed and performance, holds high practical value-especially in scenarios requiring real-time processing, such as live video analysis and online monitoring-where it can deliver fast and accurate results to meet real-world demands.

### Ablation analysis

**Table 2:** Ablation analysis.

Model	WF	SM	MAE	FPS
MSFNet-PD$_{1234}$	0.8870	0.9202	0.0174	2095.08
MSFNet-PD$_{12345}$	0.8821	0.9172	0.0180	2098.90
MSFNet-PD$_{2345}$	0.8681	0.9063	0.0204	2048.08
MSFNet-PD$_{123}$	0.8805	0.9130	0.0185	2099.16
MSFNet-PD$_{234}$	0.8799	0.9028	0.0198	2122.50
MSFNet-PD$_{345}$	0.8862	0.9187	0.0176	2092.25

Table 2 lists 6 different configurations of the MSFNet-PD model, with the differences lying in the combination of feature extractors with different dilation rates:

- **MSFNet-PD** $_{1234}$: Contains feature extractors with dilation rates of 1, 2, 3, and 4.
- **MSFNet-PD** $_{12345}$: Contains feature extractors with dilation rates of 1, 2, 3, 4, and 5.
- **MSFNet-PD** $_{2345}$: Contains feature extractors with dilation rates of 2, 3, 4, and 5.
- **MSFNet-PD** $_{123}$: Contains feature extractors with dilation rates of 1, 2, and 3.
- **MSFNet-PD** $_{234}$: Contains feature extractors with dilation rates of 2, 3, and 4.
- **MSFNet-PD** $_{345}$: Contains feature extractors with dilation rates of 3, 4, and 5.

- WF metric ranges from 0.8681 to 0.8870 across different model configurations, with the highest value being 0.8870 for MSFNet-PD$_{1234}$ and the lowest value being 0.8681 for MSFNet-PD$_{2345}$. This indicates that the model containing feature extractors with dilation rates of 1, 2, 3, and 4 performs best in this metric, while the model lacking the feature extractor with a dilation rate of 1 (MSFNet-PD$_{2345}$) performs slightly worse.
- SM metric ranges from 0.9028 to 0.9202 across different model configurations, with the highest value being 0.9202 for MSFNet-PD$_{12345}$ and the lowest value being 0.9028 for MSFNet-PD$_{234}$. This indicates that the model containing feature extractors with dilation rates of 1, 2, 3, 4, and 5 performs best in this metric, while the model lacking the feature extractor with a dilation rate of 1 (MSFNet-PD$_{234}$) performs slightly worse.
- MAE metric ranges from 0.0174 to 0.0204 across different model configurations, with the lowest value being 0.0174 for MSFNet-PD$_{1234}$ and the highest value being 0.0204 for MSFNet-PD$_{2345}$. This indicates that the model containing feature extractors with dilation rates of 1, 2, 3, and4 has the smallest error in this metric, while the model lacking the feature extractor with a dilation rate of 1 (MSFNet-PD$_{2345}$) has a slightly larger error.
- FPS metric ranges from 2048.08 to 2122.50 across different model configurations, with the highest value being 2122.50 for MSFNet-PD$_{234}$ and the lowest value being 2048.08 for MSFNet-PD$_{2345}$. This indicates that the model containing feature extractors with dilation rates of 2, 3, and 4 has the fastest speed in this metric, while the model containing feature extractors with dilation rates of 2, 3, 4, and 5 has a slightly slower speed.

From the WF, SM, and MAE metrics, the MSFNet-PD$_{1234}$ model performs the most balanced, with the best WF and MAE metrics and an SM metric close to the best value. Models containing the feature extractor with a dilation rate of 1 perform better in the WF and MAE metrics, while models containing the feature extractor with a dilation rate of 5 perform better in the SM metric. Meanwhile, the FPS metric is related to the model’s complexity, with models containing fewer feature extractors (such as MSFNet-PD$_{234}$) having faster speeds. Comparing by different model configurations, it can be found that feature extractors with different dilation rates have different impacts on model performance. For example, the feature extractor with a dilation rate of 1 has an important impact on the WF and MAE metrics, while the feature extractor with a dilation rate of 5 has an important impact on the SM metric. This analysis helps to understand the role of feature extractors with different dilation rates, thereby optimizing the model structure to achieve a better performance balance.

### Case study

**Fig. 3:**
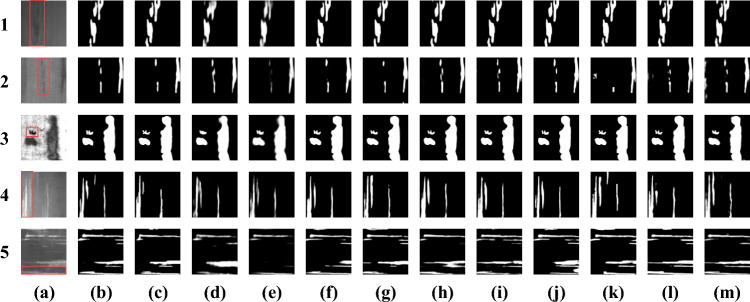
Qualitative comparisons of different models. Red boxes highlight challenging defect regions (e.g., tiny inclusions, thin scratches, and low-contrast patches). (**a**) Image, (**b**) MSFNet-PD, (**c**) BASNet, (**d**) PoolNet, (**e**) SAMNet, (**f**) U$^2$Net, (**g**) MINet, (**h**) BiSeNet, (**i**) CGNet, (**j**) EDN, (**k**) EDRNet, (**l**) F$^3$Net, (**m**) LEDNet.

Figure 3 presents a comprehensive qualitative comparison of different salient object detection (SOD) models applied to the surface defect detection task. From the visualization results, it is evident that the proposed MSFNet-PD achieves defect localization and segmentation performance that is highly competitive with the recent representative methods, MINet. In the red-boxed regions shown in Fig. 3, which include representative defect types such as tiny inclusions, thin and elongated scratches, and low-contrast patches, MSFNet-PD preserves structural continuity and suppresses background interference more effectively than most competing models. Both models demonstrate excellent performance in terms of accurately identifying defect regions and delineating their boundaries. Specifically, the defect areas predicted by MSFNet-PD exhibit high structural integrity and boundary clarity, closely matching the ground truth without significant false positives or false negatives. These results confirm that MSFNet-PD can maintain high precision and recall under varying defect shapes, sizes, and surface complexities-indicating its robustness and adaptability in real-world industrial scenarios.

Notably, although BASNet shares a comparable parameter scale with MSFNet-PD, its detection performance is visibly poorer. The defect regions predicted by BASNet are often incomplete, blurred, or fragmented, with some background areas mistakenly detected as defects. This suggests that BASNet is less capable of capturing discriminative semantic features and fine-grained spatial details crucial for accurate defect detection. In contrast, MSFNet-PD demonstrates superior structural design and feature extraction capabilities, largely attributed to its multi-scale fusion mechanism and the use of progressive dilated convolutions. These architectural components enable the model to integrate multi-level contextual information while maintaining spatial resolution, thereby enhancing its ability to capture both coarse and fine patterns associated with surface anomalies.

Specifically, the defect areas predicted by MSFNet-PD exhibit high structural integrity and boundary clarity. Taking the defect in the first row of Figure 3 as an example, MSFNet-PD achieves a Weighted F-measure (WF) of **0.8870**, which achieves competitive performance compared with BiSeNet (**0.8803**) and is **highly competitive** with other lightweight models like MINet (Re-implement) (**0.8873**). Furthermore, MSFNet-PD also demonstrates competitive performance to many heavy-parameter models like BASNet (although **0.9092** is higher, MSFNet-PD achieves much higher speed). These quantitative results, combined with visual inspections, confirm that MSFNet-PD achieves excellent defect localization and segmentation performance while maintaining a significantly high inference speed (**2095** FPS) and a lightweight parameter budget (**1.59M**).

Furthermore, the improved defect localization accuracy achieved by MSFNet-PD-despite maintaining a lightweight parameter budget-reflects its efficiency in parameter utilization and architectural optimization. This balance between detection accuracy and model complexity is critical for deployment in real-time quality inspection systems on production lines, where both precision and inference speed are equally essential.

In summary, the qualitative results depicted in Fig. 3 not only demonstrate the strong qualitative performance of MSFNet-PD over conventional models like BASNet but also validate its strong competitiveness against cutting-edge models like MINet. These observations highlight the practical effectiveness and theoretical soundness of MSFNet-PD in addressing the challenges of real-time surface defect detection.

## Conclusion

In this paper, we presented MSFNet-PD, a novel and efficient framework for real-time salient object detection of surface defects on strip steel. The proposed model integrates a multi-scale feature fusion architecture with a progressive dilation strategy, enabling it to effectively capture both fine-grained local textures and high-level semantic structures of diverse defect patterns. By gradually increasing dilation rates across convolutional layers, the network adaptively expands its receptive field without incurring additional computational burden or compromising resolution. To further ensure real-time applicability in industrial scenarios, MSFNet-PD adopts a lightweight backbone and an efficient feature fusion module, significantly improving inference speed while maintaining high detection accuracy. Extensive experiments on the SD-Saliency-900 dataset demonstrate that MSFNet-PD achieves competitive performance compared to existing methods, validating its effectiveness and efficiency in practical defect inspection environments. In future work, we plan to explore the integration of transformer-based global context modeling with our multi-scale fusion strategy to further improve the detection of subtle and irregular defects. Additionally, we aim to extend MSFNet-PD to other types of industrial surfaces and edge-computing platforms to enhance its generalizability and deployment scalability.Although MSFNet-PD demonstrates strong real-time performance on SD-Saliency-900, its generalization to broader industrial datasets requires further validation. Future work will explore cross-domain evaluation and additional real-world scenarios.

## Data Availability

The datasets used in this study are publicly available. The SD-Saliency-900 dataset can be accessed at: https://github.com/SongGuorong/MCITF/tree/master/SD-Saliency-900.
